# Prenatal Arsenic Exposure and Birth Outcomes among a Population Residing near a Mining-Related Superfund Site

**DOI:** 10.1289/ehp.1510070

**Published:** 2016-02-09

**Authors:** Birgit Claus Henn, Adrienne S. Ettinger, Marianne R. Hopkins, Rebecca Jim, Chitra Amarasiriwardena, David C. Christiani, Brent A. Coull, David C. Bellinger, Robert O. Wright

**Affiliations:** 1 Department of Environmental Health, Boston University School of Public Health, Boston, Massachusetts, USA; 2 Department of Nutritional Sciences, University of Michigan School of Public Health, Ann Arbor, Michigan, USA; 3 Department of Environmental Health, Harvard T.H. Chan School of Public Health, Boston, Massachusetts, USA; 4 Local Environmental Action Demanded (L.E.A.D.) Agency, Inc., Vinita, Oklahoma, USA; 5 Division of Environmental Health, Icahn School of Medicine at Mount Sinai, New York, New York, USA; 6 Department of Biostatistics, Harvard T.H. Chan School of Public Health, Boston, Massachusetts, USA; 7 Department of Neurology, and; 8 Department of Psychiatry, Harvard Medical School and Boston Children’s Hospital, Boston, Massachusetts, USA

## Abstract

**Background:**

Limited epidemiologic data exist on prenatal arsenic exposure and fetal growth, particularly in the context of co-exposure to other toxic metals.

**Objective:**

We examined whether prenatal arsenic exposure predicts birth outcomes among a rural U.S. population, while adjusting for exposure to lead and manganese.

**Methods:**

We collected maternal and umbilical cord blood samples at delivery from 622 mother–infant pairs residing near a mining-related Superfund site in Northeast Oklahoma. Whole blood arsenic, lead, and manganese were measured using inductively coupled plasma mass spectrometry. We modeled associations between arsenic concentrations and birth weight, gestational age, head circumference, and birth weight for gestational age.

**Results:**

Median (25th–75th percentile) maternal and umbilical cord blood metal concentrations, respectively, were as follows: arsenic, 1.4 (1.0–2.3) and 2.4 (1.8–3.3) μg/L; lead, 0.6 (0.4–0.9) and 0.4 (0.3–0.6) μg/dL; manganese, 22.7 (18.8–29.3) and 41.7 (32.2–50.4) μg/L. We estimated negative associations between maternal blood arsenic concentrations and birth outcomes. In multivariable regression models adjusted for lead and manganese, an interquartile range increase in maternal blood arsenic was associated with –77.5 g (95% CI: –127.8, –27.3) birth weight, –0.13 weeks (95% CI: –0.27, 0.01) gestation, –0.22 cm (95% CI: –0.42, –0.03) head circumference, and –0.14 (95% CI: –0.24, –0.04) birth weight for gestational age z-score units. Interactions between arsenic concentrations and lead or manganese were not statistically significant.

**Conclusions:**

In a population with environmental exposure levels similar to the U.S. general population, maternal blood arsenic was negatively associated with fetal growth. Given the potential for relatively common fetal and early childhood arsenic exposures, our finding that prenatal arsenic can adversely affect birth outcomes is of considerable public health importance.

**Citation:**

Claus Henn B, Ettinger AS, Hopkins MR, Jim R, Amarasiriwardena C, Christiani DC, Coull BA, Bellinger DC, Wright RO. 2016. Prenatal arsenic exposure and birth outcomes among a population residing near a mining-related Superfund site. Environ Health Perspect 124:1308–1315; http://dx.doi.org/10.1289/ehp.1510070

## Introduction

Size at birth is an important predictor of early childhood survival and morbidity, and a predictor of chronic diseases in adulthood. Factors influencing fetal growth are numerous, and include maternal health and nutritional status. Maternal exposure to environmental toxicants, including tobacco smoke, air pollution, and metals, also predicts birth outcomes (Wigle et al. 2007).

Metals, including lead (Pb), manganese (Mn), and inorganic arsenic (As), readily cross the placenta (Concha et al. 1998; Graziano et al. 1990; Krachler et al. 1999), exposing the fetus. Prenatal exposure to these metals is associated with lower birth weight (Chen et al. 2014; Kordas et al. 2009; Quansah et al. 2015), shorter gestation (Xu et al. 2011; Yang et al. 2003), and smaller chest and head circumference (Guan et al. 2014; Rahman et al. 2009). Previous birth outcomes studies were conducted mostly in areas of unusally high arsenic or lead exposure, among populations that also may have been at increased risk of poor overall health and nutritional status (Huyck et al. 2007; Xu et al. 2012; Zheng et al. 2014). Few birth outcomes studies have examined associations with low to moderate levels of metal exposures that are consistent with background levels in many U.S. communities (Fei et al. 2013; Shi et al. 2015). Research on environmental lead exposure has demonstrated adverse effects at exposure concentrations much lower than in highly exposed populations (Xie et al. 2013). A similar phenomenon may be occurring for other common environmental contaminants, such as arsenic, for which most research has focused on high exposure levels, as was historically the case for lead. Recent reviews of the literature on arsenic exposure and birth outcomes called for additional epidemiologic studies with individual-level data to address the data gap in populations exposed to lower arsenic levels (Bloom et al. 2014; Quansah et al. 2015). Furthermore, exposures do not occur in isolation, and exposure to multiple metals may more accurately reflect real-world exposure scenarios (Hu et al. 2007). Metals often co-occur in the environment, especially near Superfund sites (Hu et al. 2007; Tchounwou et al. 2012), and studies have reported positive correlations between arsenic, lead, and manganese in biomarkers (Calderón et al. 2001; Delves et al. 1973; Joselow et al. 1978) and in environmental media (Zota et al. 2011). We are not aware of any study that has looked at associations between arsenic and birth outcomes, adjusting for lead and manganese. Given each metal’s potential to be independently associated with birth outcomes, and the prevalence of concurrent exposures, adjustment for co-occurring metal exposures is important. Additionally, metals may act synergistically or antagonistically to affect birth outcomes; yet metal interactive effects have not previously been reported.

In this study we examined associations between prenatal arsenic exposure and birth outcomes, while simultaneously adjusting for lead and manganese, among mother–infant pairs residing in Northeast Oklahoma. Despite being located near a mining-related Superfund site, this area has relatively low levels of arsenic and lead in environmental media (Zota et al. 2011). We previously reported a nonlinear, inverted-U shaped association of prenatal manganese exposure with birth weight in this population (Zota et al. 2009); here, we expand this work to arsenic.

## Methods

### Study Subjects

Subjects were participants in a prospective birth cohort study (Metals Assessment Targeting Community Health, “MATCH”) of biologic markers of prenatal and early childhood exposure to metals, maternal psychosocial stress, and their impacts on neurodevelopment. This cohort was enrolled in the area surrounding the Tar Creek Superfund site in Ottawa County, Oklahoma. Details of the study including location and objectives have been described elsewhere (Ettinger et al. 2009; Zota et al. 2009). Briefly, pregnant women were recruited during prenatal visits or at delivery from the Integris Baptist Regional Health Center (Miami, OK), the only birthing facility in the county. Eligibility criteria included *a*) giving birth at Integris Hospital; *b*) intention to live within the study area for the next 2 years; *c*) not currently enrolled in the study with another child; and *d*) English-language proficiency sufficient to participate in the informed consent process. Eligible mothers received a detailed explanation of study procedures before consenting to participate. The research protocol was approved by the Human Subjects Committees of Integris Health and Harvard School of Public Health (HSPH).

Between 2002 and 2011, 1,996 individuals were screened, of whom 1,322 met inclusion criteria. Of these, 713 mother–infant pairs were enrolled in MATCH. For the present study, we excluded children with very low birth weights (i.e., < 1,500 g; *n* = 1), multiple births (*n* = 7), and those for whom data were missing on birth weight (*n* = 1), gestational age (*n* = 3), or all metal biomarker levels (*n* = 2). Metal biomarker data were excluded on 70 additional mother–infant pairs to avoid batch effects inherent in different instruments due to our laboratory’s purchase of a new inductively coupled plasma mass spectrometry (ICP-MS) instrument near the end of the study, leaving 622 mother–infant pairs for this analysis.

### Exposure Assessment

Maternal blood and umbilical cord blood samples were collected at delivery (± 12 hr) and analyzed for total arsenic concentration at the Trace Metals Laboratory at HSPH (Boston, MA). Blood lead and manganese concentrations were also measured and considered as covariates in all analyses. Venous whole blood was collected in trace element–free tubes (BD Vacutainer royal blue top, with K2EDTA #368381; Becton Dickinson, Franklin Lakes, NJ) and shipped frozen to the laboratory. Blood (1 mL) was digested with concentrated HNO_3_ acid, followed by addition of hydrogen peroxide and dilution with deionized water. Total arsenic, lead, and manganese concentrations were measured with a dynamic reaction cell/inductively coupled plasma mass spectrometer (DRC/ICP-MS; Elan 6100; PerkinElmer, Norwalk, CT) using previously published methods and quality control procedures (Chen et al. 1999; Ettinger et al. 2009). Average recovery of quality control standards was 75–110%. The limit of detection (LOD) was 0.02 μg/dL for blood arsenic, lead, and manganese. Two (0.3%) arsenic and three (0.5%) lead measurements in cord blood were below the LOD, for which we assigned a value of half the LOD.

### Birth Outcomes and Covariates

Data on the four birth outcomes examined in the MATCH study (birth weight, birth length, head circumference, and gestational age at birth) were abstracted from medical records by study staff who were blind to exposure biomarker levels. Birth weight, length, and head circumference were measured by delivery room staff using standard clinical procedures. Gestational age at birth was based on clinical assessment using data from the last menstrual period, the first accurate ultrasound examination during the first trimester, and clinical examination (ACOG 2014).

Information on maternal pregnancy health and prenatal care such as glucose challenge test results was obtained from medical records. Interviewer-administered questionnaires were used to collect information on potential sources of metals exposure, prepregnancy weight and demographic and social characteristics such as mother’s birth date, marital status, race/ethnicity, smoking status, and prenatal vitamin use.

### Statistical Analysis

Univariate and bivariate summary statistics and distributional plots were generated for all variables. Spearman correlations were calculated among exposure biomarkers. Distributions of metal concentrations were positively skewed; therefore, we used natural log-transformed metals in all models.

Associations between arsenic and four birth outcomes were estimated: birth weight (g), gestational age (weeks), head circumference (cm), and birth weight for gestational age (*z*-score). Weight for gestational age, an estimate of fetal growth, was calculated based on the median birth weight for each completed week of gestation for a 1999–2000 U.S. natality data set (Oken et al. 2003). Weight for gestational age was then converted to a normal *z*-value, where each unit represents the distance of birth weight from population median for a given gestational age scaled by population standard deviation (Oken et al. 2004).

To estimate associations between arsenic and birth outcomes, we used semiparametric regression that allows for possible nonlinearity in associations between covariates and birth outcomes. All models included arsenic, lead, and manganese (lead and manganese centered at mean of log_e_ distribution) as well as an *a priori* set of covariates consistently associated with fetal growth parameters in the literature: maternal age (years) at child’s birth, infant sex, race/ethnicity [white, Native American, other (African American or black, Asian, Native Hawaiian, Pacific Islander)], primiparity, and maternal smoking during pregnancy. Additional maternal characteristics considered as potential confounders were: pre-pregnancy body mass index (BMI; kg/m^2^), gestational weight gain (kg), blood (plasma) glucose (mg/dL) measured 1 hr after a 50 g oral glucose challenge between 24 and 28 weeks gestation (as part of routine prenatal care), hemoglobin (g/dL) at delivery, education (≥ 12th vs. < 12th grade), marital status (married/living with partner vs. not), any smokers in the home during pregnancy and prenatal vitamin use. Inclusion of covariates in final multivariable models was based on *a*) covariate associations with biomarkers and birth outcomes in bivariate models (α = 0.1), *b*) model fit (partial *F*-test at α = 0.1), and *c*) change in arsenic effect estimates (> 10%). All final multivariable models were adjusted for blood lead and manganese, infant sex, maternal age at child’s birth, race/ethnicity, parity, smoking during pregnancy, education, prenatal vitamin use, and hemoglobin. We modeled lead and manganese as smoothed terms using generalized additive models (GAMs) with penalized splines, given previous evidence of nonlinear associations with birth weight (Zhu et al. 2010; Zota et al. 2009). Maternal age at child’s birth was modeled as a smoothed term because visual assessment of exploratory smoothed plots suggested deviations from linearity. We performed complete case analyses and assumed data were missing at random.

To obtain effect estimates for arsenic, we modeled arsenic concentrations as *a*) quartiles, with the lowest quartile as the referent group, and *b*) continuous log_e_-transformed concentrations to compare the 75th with the 25th percentile (interquartile range increase). Birth outcomes were modeled as continuous variables. To assess maternal and cord blood arsenic as two different proxies of prenatal exposure and to compare their ability to predict birth outcomes, we additionally fit models including both maternal and cord blood arsenic simultaneously. A *p*-value < 0.05 was considered statistically significant. We explored arsenic interactions with lead and manganese on birth outcomes by including cross-product terms of log_e_-transformed metals in the models (i.e., arsenic × lead, arsenic × manganese). Interaction cross-product terms were considered to be statistically significant at *p* < 0.1.

Because of our previous finding in this cohort of a positive association between maternal blood arsenic and impaired glucose tolerance during pregnancy (Ettinger et al. 2009), we evaluated impaired glucose tolerance as a potential confounder and effect modifier of arsenic–birth outcomes associations. To evaluate potential confounding, regression models were adjusted for impaired glucose tolerance, defined as blood glucose level ≥ 140 mg/dL (vs. < 140 mg/dL) measured 1 hr after a 50-g glucose challenge, which is used as standard screening criteria to identify women who should receive further testing for gestational diabetes (Vandorsten et al. 2013). To evaluate interaction, we used two approaches: *a*) Models were stratified by impaired glucose tolerance status, and *b*) models included a cross-product term between arsenic and impaired glucose tolerance.

Arsenic may influence fetal development in a sex-dependent manner (Kippler et al. 2012; Xu et al. 2011). Therefore, we assessed sex differences by stratifying analyses and by evaluating a sex-arsenic cross-product term.

Sensitivity analyses assessed the robustness of the observed associations to evaluate *a*) the extent of confounding by co-occurring metals, by excluding lead and manganese from models but adjusting for other covariates; *b*) arsenic effects on head circumference independent of gestational length, by including gestational age at birth as a covariate; and *c*) the extent of exposure measurement error, which may be particularly important when examining biomarkers measured in the same medium (Pollack et al. 2013), by weighting models with the inverse of the arsenic measurement error (exposure) variance of the five laboratory replicates per sample (Cochran and Carroll 1953). This inverse variance weighting approach assigns greater weight to measurements with low uncertainties (Bevington and Robinson 2003). We used SAS version 9.4 (SAS Institute, Inc., Cary, NC) and R version 3.1.2 (The R Foundation for Statistical Computing, www.r-project.org) (R Core Team 2008).

## Results

Sociodemographic characteristics and birth outcomes are summarized in Table 1. Included mother–infant pairs (*n* = 622) were similar on most characteristics to subjects who were excluded from analyses (*n* = 91; Table 1). Compared with excluded mother–infant pairs, included pairs more frequently reported smoking during pregnancy (*p* = 0.03) and taking prenatal vitamins (*p* < 0.0001). Data were not available on certain characteristics (Table 1), primarily because information was missing from medical records or participants may have chosen to omit information related to certain family characteristics, potential sources of exposure, or psychosocial stress during questionnaire administration by trained interviewers. Of 622 mother–infant pairs, data were not available for 4 (0.6%) maternal and 13 (2%) cord blood arsenic measurements, 3 (0.5%) cord blood lead and manganese measurements, and 24 (3.9%) head circumference measurements at birth.

**Table 1:** Characteristics of mother–infant pairs [*n* (%) or mean ± SD].

Characteristic	Included participants (*n* = 622)^*a*^	Excluded participants (*n* = 91)^*b*^
Maternal
Age at delivery (years)	24.5 ± 5.5	24.5 ± 5.2
Prepregnancy BMI (kg/m^2^)	26.9 ± 6.4	28.8 ± 7.0
Weight gain during pregnancy (kg)	13.1 ± 7.2	13.6 ± 7.7
Marital status
Married or living with partner	391 (64.1)	64 (73.6)
Never married/separated/divorced	219 (35.9)	23 (26.4)
Race/ethnicity
White	408 (67.2)	58 (67.4)
Native American	143 (23.6)	24 (27.9)
Other (including Hispanic)	56 (9.2)	4 (4.7)
Education
< 12th grade	159 (25.6)	25 (28.1)
≥ 12th grade	462 (74.4)	64 (71.9)
Annual household income
< $20,000	188 (47.5)	45 (60.0)
$20,000–$40,000	134 (33.8)	19 (25.3)
$40,000–$70,000	61 (15.4)	7 (9.3)
> $70,000	13 (3.3)	4 (5.3)
Impaired glucose tolerance, 24–28 weeks gestation
< 140 mg/dL	508 (87.1)	65 (84.4)
≥ 140 mg/dL	75 (12.9)	12 (15.6)
Primiparous
Yes	243 (39.1)	32 (35.6)
No	378 (60.9)	58 (64.4)
Smoked during pregnancy*
Yes	224 (36.0)	22 (24.4)
No	398 (64.0)	68 (75.6)
Any smokers in household
Yes	134 (38.0)	32 (40.5)
No	219 (62.0)	47 (59.5)
Prenatal vitamin use*
Yes	397 (63.8)	32 (35.2)
No	225 (36.2)	59 (64.8)
Anemia at delivery^*c*^
Yes	156 (25.3)	19 (22.4)
No	460 (74.7)	66 (77.6)
Hemoglobin at delivery (g/dL)	11.8 ± 1.4	11.7 ± 1.2
Infant
Birth weight (g)	3370.2 ± 474.5	3245.6 ± 462.3
Gestational age at birth (week)	39.1 ± 1.3	38.7 ± 1.9
Length at birth (cm)	50.0 ± 2.6	50.8 ± 2.4
Head circumference at birth (cm)	34.5 ± 1.8	34.1 ± 1.3
Male sex	340 (54.7)	46 (51.7)
^***a***^Of included participants, data were missing for prepregnancy BMI (*n *= 63), weight gain during pregnancy (*n *= 63), marital status (*n *= 12), race/ethnicity (*n *= 15), education (*n *= 1), household income (*n *= 226), impaired glucose tolerance (*n *= 39), primiparous (*n *= 1), any smokers in household (*n *= 269), anemia at delivery (*n *= 6), hemoglobin at delivery (*n *= 6), length at birth (*n *= 10), and head circumference at birth (*n *= 24). ^***b***^Of excluded participants, data were missing for age at delivery (*n *= 3), prepregnancy BMI (*n *= 6), weight gain during pregnancy (*n *= 5), marital status (*n *= 4), race/ethnicity (*n *= 5), education (*n *= 2), household income (*n *= 16), impaired glucose tolerance (*n *= 14), primiparous (*n *= 1), smoked during pregnancy (*n *= 1), any smokers in household (*n *= 12), anemia (*n *= 6), hemoglobin at delivery (*n *= 6), birth weight (*n *= 3), gestational age at birth (*n *= 6), length at birth (*n *= 6), head circumference at birth (*n *= 7), and male sex (*n *= 2). ^***c***^Anemia is defined as hemoglobin < 11.0 g/dL at delivery, which is based on definition from CDC (1998; during 3rd trimester), WHO (1968; during pregnancy), and the American Congress of Obstetricians and Gynecologists (2008; in 1st and 3rd trimesters). *Participants differed from nonparticipants, *p* < 0.05 in chi-square test.		

Metals concentrations in maternal and infant biomarkers are presented in Table 2. Median (25th–75th percentile) maternal and umbilical cord blood metal concentrations, respectively, were, for arsenic, 1.4 (0.97–2.3) and 2.4 (1.8–3.3) μg/L; lead, 0.60 (0.41–0.88) and 0.43 (0.28–0.62) μg/dL; manganese, 22.7 (18.8–29.3) and 41.7 (32.2–50.4) μg/L. Maternal blood concentrations were correlated with cord blood concentrations for arsenic, *r* = 0.35 (*p* < 0.001); lead, *r* = 0.75 (*p* < 0.001); and manganese, *r* = 0.39 (*p* < 0.001) (Table 2). In maternal blood, weak correlations with arsenic were observed for lead (*r* = 0.10, *p* = 0.01) and manganese (*r* = 0.11, *p* = 0.01). Manganese and lead were also weakly correlated (*r* = 0.16, *p* < 0.001). In cord blood, correlations with lead were observed for arsenic (*r* = 0.18, *p* < 0.001) and manganese (*r* = 0.23, *p* < 0.001). Cord blood manganese was significantly higher among boys than girls [geometric mean (geometric standard deviation)]: boys, 41.1 (1.5); girls, 37.4 (1.5) μg/L, *p* = 0.004). No significant sex differences were observed in other biomarkers.

**Table 2:** Maternal and infant biomarkers.

Biomarker	Maternal blood			Umbilical cord blood			Corr^*a*^
	*n*	Median (25th–75th percentile)	Range	*n*	Median (25th–75th percentile)	Range	
As (μg/L)	618	1.4 (0.97–2.3)	0.23–24.1	609	2.4 (1.8–3.3)	< LOD^*b*^–13.2	0.35*
Pb (μg/dL)	622	0.60 (0.41–0.88)	0.03–3.1	619	0.43 (0.28–0.62)	< LOD^*b*^–3.9	0.75*
Mn (μg/L)	622	22.7 (18.8–29.3)	8.0–117.4	619	41.7 (32.2–50.4)	5.4–139.1	0.39*
^***a***^Spearman’s correlation (Corr) between maternal and infant blood. ^***b***^Limit of detection is 0.02 μg/dL. *p < 0.001.							

Bivariate analyses with arsenic modeled as a continuous outcome variable (log_e_ scale) showed that prenatal vitamin use was associated with lower maternal (28%) and cord (15%) blood arsenic (maternal: β = –0.33; 95% CI: –0.44, –0.23; cord: β = –0.16; 95% CI: –0.27, –0.06). Primiparous mothers had 12% lower arsenic than multiparous women (β = –0.13; 95% CI: –0.24, –0.03). Compared to white mothers, Native American mothers had 14% higher (β = 0.13; 95% CI: 0.01, 0.26) and other ethnicities had 17% lower (β = –0.19; 95% CI: –0.38, –0.01) arsenic levels. Maternal characteristics associated with significantly (*p* < 0.05) higher maternal arsenic were higher age at delivery, higher blood glucose challenge levels, lower hemoglobin at delivery, education (≥ 12th grade) and being married/living with partner (data not shown). Fewer significant associations were observed between cord blood arsenic and covariates.

**Figure 1:**
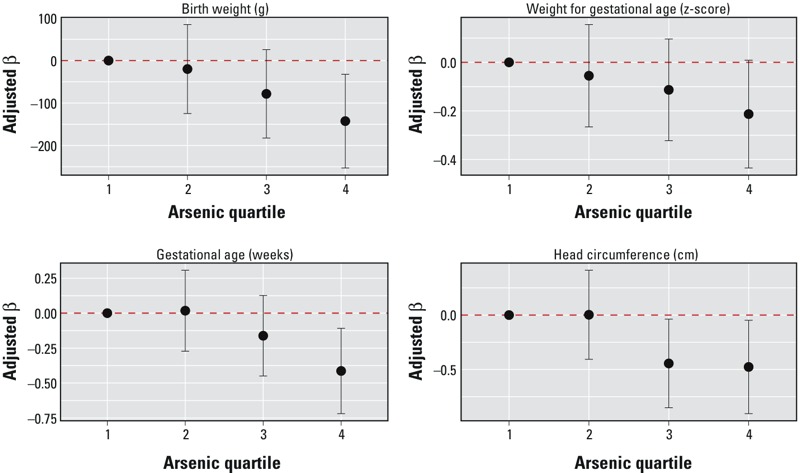
Effect estimates and 95% confidence intervals for quartiles of maternal blood arsenic with birth weight (BW), gestational age (GA), weight for gestational age (BW for GA), and head circumference (HC). Generalized additive models include maternal blood lead and manganese (smoothed), maternal age at delivery (smoothed), infant sex, race/ethnicity, parity, smoking during pregnancy, maternal education, prenatal vitamin use, and maternal hemoglobin at delivery (*n *= 596 for BW, GA, BW for GA; *n *= 574 for HC). Dashed horizontal line represents null association.

In multivariable models, maternal blood arsenic was consistently negatively associated with all outcomes. Adjusted effect estimates and 95% CIs for all birth outcomes across quartiles of maternal blood arsenic are presented in Figure 1. Compared with low exposure (quartile 1), high maternal arsenic exposure (quartile 4) was associated with significantly lower birth weight (β_Q4_ = –142.5; 95% CI: –252.9, –32.2), shorter gestational length (β_Q4_ = –0.41; 95% CI: –0.72, –0.11), smaller head circumference (β_Q4_ = –0.48; 95% CI: –0.91, –0.05), and lower birth weight for gestational age *z*-scores (β_Q4_ = –0.21; 95% CI: –0.43, 0.01) (see Table S1). An interquartile range increase in arsenic was associated with –77.5 g (95% CI: –127.8, –27.3) birth weight; –0.13 weeks (95% CI: –0.27, 0.01) gestation; –0.22 cm (95% CI: –0.42, –0.03) head circumference; and –0.14 (95% CI: –0.24, –0.04) weight for gestational age *z*-scores (see Table S1).

**Figure 2:**
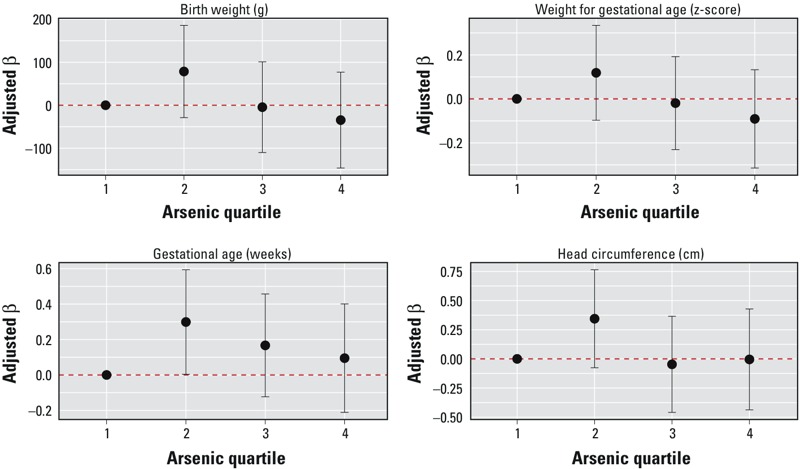
Effect estimates and 95% confidence intervals for quartiles of cord blood arsenic with birth weight (BW), gestational age (GA), weight for gestational age (BW for GA), and head circumference (HC). Generalized additive models include cord blood lead and manganese (smoothed), maternal age at delivery (smoothed), infant sex, race/ethnicity, parity, smoking during pregnancy, maternal education, prenatal vitamin use, and maternal hemoglobin at delivery (*n *= 588 for BW, GA, BW for GA; *n *= 565 for HC). Dashed horizontal line represents null association.

Associations between cord blood arsenic and birth outcomes were less apparent. Figure 2 presents adjusted effect estimates and 95% CIs across quartiles of cord blood arsenic with all birth outcomes. Compared with low levels (quartile 1), high cord arsenic levels (quartile 4) were associated with lower birth weight (β_Q4_ = –34.6; 95% CI: –146.1, 76.9) and birth weight for gestational age *z*-scores (β_Q4_ = –0.09; 95% CI: –0.31, 0.13), but these associations were not statistically significant (see Table S1). There were positive associations between cord blood arsenic (quartiles) and gestational age, though the association was only statistically significant in quartile 2 (vs. quartile 1, β_Q2_ = 0.30; 95% CI: 0.00, 0.59). There was no difference in fit between models with cord arsenic parameterized as quartiles and as continuous log_e_-transformed concentrations (likelihood ratio tests: *p* > 0.05 for all birth outcomes) and no significant association between continuous cord arsenic and birth outcomes (see Table S1).

In models including both maternal and cord blood arsenic, effect estimates for cord arsenic were similar while estimates for maternal arsenic were attenuated (data not shown), but remained negative and statistically significant for most relationships (e.g., –58.9; 95% CI: –114.5, –3.2 g birth weight for interquartile range increase in maternal arsenic, –0.38; 95% CI: –0.71, –0.06 weeks gestation for quartile 4 vs. quartile 1, –0.23; 95% CI: –0.45, –0.01 cm head circumference for interquartile range increase). No significant interactions were observed between maternal or cord blood arsenic with lead or manganese (see Table S2). When we included a cross-product term between arsenic and sex, the interaction terms were not significant for maternal or cord blood arsenic with any birth outcomes (e.g., birth weight: maternal As × sex, *p* = 0.6; data not shown).

Given our previous report in which blood arsenic was associated with impaired glucose tolerance during pregnancy (Ettinger et al. 2009), we examined impaired glucose tolerance (≥ 140 mg/dL vs. < 140 mg/dL; prevalence of impaired glucose tolerance = 12.9%) as a potential confounder and effect modifier of arsenic–birth outcomes associations. Adjusting for impaired glucose tolerance changed maternal and cord blood arsenic effect estimates minimally (e.g., for interquartile range increase in maternal arsenic, with adjustment: –72.8; 95% CI: –124.2, –21.4 g birth weight; without: –77.5; 95% CI: –127.8, –27.3 g; data not shown), suggesting that impaired glucose tolerance status is not a strong confounder of the arsenic–birth outcomes associations. In these models, impaired glucose tolerance was positively associated with weight for gestational age (β = 0.25; 95% CI: 0.01, 0.49 *z*-scores); no other associations were observed between impaired glucose tolerance and birth outcomes (data not shown). Glucose tolerance status may also be a causal intermediate between arsenic and birth outcomes. Maternal arsenic was positively associated with impaired glucose tolerance (*p* = 0.007); however, the small changes in arsenic estimates when conditioning on glucose intolerance suggest that impaired glucose tolerance may only be a weak, partial mediator of the arsenic–birth outcomes associations. To evaluate effect modification, we stratified by impaired glucose tolerance status. Compared with women with normal post-challenge glucose levels, those with high glucose had steeper slopes for arsenic with all birth outcomes, though confidence intervals were wider (Table 3). In models including cross-product terms between arsenic and impaired glucose tolerance status, there was a significant negative interaction between maternal arsenic and glucose tolerance status on gestational age (e.g., *p* = 0.04 for cross-product term between continuous arsenic and impaired glucose tolerance; Table 3), suggesting that high arsenic exposure is associated with accelerated shortened gestational length in the presence of abnormal glucose tolerance.

**Table 3:** Adjusted associations*^a^* of maternal blood arsenic with birth outcomes, stratified by maternal glucose tolerance status.

Outcome and exposure	Normal (< 140 mg/dL)		Impaired glucose tolerance (≥ 140 mg/dL)		*p*-Value for interaction^*c*^
	*n*	Estimate (95% CI)^*b*^	*n*	Estimate (95% CI)^*b*^	
Birth weight
As quartile 1^*d*^	132	0 (reference)	11	0 (reference)
Quartile 2	120	–21.0 (–134.2, 92.2)	13	–409.0 (–771.7, –46.3)	0.33
Quartile 3	118	–112.9 (–228.3, 2.6)	24	–183.5 (–495.3, 128.3)	0.76
Quartile 4	115	–115.3 (–235.6, 5.1)	24	–562.6 (–919.6, –205.7)	0.20
Per IQR increase in As	485	–68.6 (–123.4, –13.8)	72	–185.0 (–358.6, –11.4)	0.62
Gestational age
As quartile 1	132	0 (reference)	11	0 (reference)
Quartile 2	120	0.00 (–0.31, 0.32)	13	–0.33 (–1.4, 0.75)	0.70
Quartile 3	118	–0.12 (–0.44, 0.20)	24	–0.74 (–1.7, 0.22)	0.49
Quartile 4	115	–0.33 (–0.66, 0.00)	24	–1.7 (–2.8, –0.60)	0.06*
Per IQR increase in As	485	–0.07 (–0.22, 0.08)	72	–0.73 (–1.2, –0.23)	0.04*
Birth weight for gestational age
As quartile 1	132	0 (reference)	11	0 (reference)
Quartile 2	120	–0.05 (–0.28, 0.18)	13	–0.81 (–1.5, –0.09)	0.28
Quartile 3	118	–0.20 (–0.43, 0.03)	24	–0.10 (–0.73, 0.52)	0.54
Quartile 4	115	–0.18 (–0.42, 0.07)	24	–0.70 (–1.4, 0.02)	0.47
Per IQR increase in As	485	–0.14 (–0.25, –0.03)	72	–0.21 (–0.56, 0.14)	0.84
Head circumference
As quartile 1	127	0 (reference)	11	0 (reference)
Quartile 2	113	–0.02 (–0.47, 0.43)	13	–1.2 (–2.5, 0.04)	0.63
Quartile 3	113	–0.58 (–1.0, –0.13)	23	–0.75 (–1.8, 0.35)	0.67
Quartile 4	112	–0.44 (–0.91, 0.04)	24	–1.9 (–3.1, –0.65)	0.58
Per IQR increase in As	465	–0.21 (–0.43, 0.01)	71	–0.67 (–1.3, –0.08)	0.87
^***a***^Models were adjusted for maternal blood Pb and Mn (smoothed), maternal age at child’s birth (smoothed), infant sex, race/ethnicity, parity, smoking during pregnancy, maternal education, prenatal vitamin use, and maternal hemoglobin at delivery. ^***b***^Effect estimates represent change in birth outcomes for arsenic quartiles 2, 3, 4 compared to quartile 1; or interquartile range increase in arsenic (continuous log_e_-transformed concentrations, scaled to the interquartile range. ^***c***^Interaction cross-product term between arsenic and impaired glucose tolerance. ^***d***^Arsenic quartile 1: < 0.97 μg/L, quartile 2: ≥ 0.97 to < 1.4 μg/L, quartile 3: ≥ 1.4 to < 2.3 μg/L, quartile 4: ≥ 2.3 μg/L. *Interaction *p*-value < 0.10.					

We fit models without lead and manganese to examine the extent of confounding by co-occurring metals (data not shown). Arsenic effect estimates were similar to those from models with lead and manganese. The main difference was found for birth weight with slightly attenuated arsenic estimates (e.g., for interquartile range increase in maternal arsenic, without lead and manganese: –68.8; 95% CI: –118.5, –19.0 g; with: –77.5; 95% CI: –127.8, –27.3 g). When we additionally adjusted for gestational age in models of head circumference, associations for maternal arsenic weakened (for interquartile range increase in maternal arsenic, with gestational age: –0.16; 95% CI: –0.35, 0.03 cm; without: –0.22; 95% CI: –0.42, –0.03 cm). In models accounting for measurement error (weighted by the inverse of arsenic measurement error variance), arsenic effect estimates changed little (see Table S3).

## Discussion

In this U.S. population, arsenic concentrations in maternal and cord blood were lower relative to studies conducted in Asia and South America (Ahmed et al. 2012; Concha et al. 1998; Guan et al. 2012), where naturally occurring geologic factors likely caused increased arsenic exposures from drinking water. Compared with other populations with no known source of high arsenic exposure in the United States (Sanders et al. 2012) and Europe (Remy et al. 2014), blood arsenic levels near Tar Creek are slightly higher, perhaps reflecting exposure from nearby mining waste: geometric mean cord blood = 0.56 μg/L (Remy et al. 2014), third-trimester maternal blood = 0.44 μg/L (Sanders et al. 2012) vs. Tar Creek cord blood = 2.3 μg/L and maternal blood = 1.5 μg/L. Even with lower-level exposures, we observed decreases in birth weight, fetal growth, gestational length, and head circumference with increasing prenatal arsenic exposure while adjusting for lead and manganese. This is among the first studies of metals and birth outcomes to consider metal co-exposures as potential confounders and effect modifiers. Simultaneous exposure to multiple metals is a realistic exposure scenario, particularly in areas such as this Superfund site (Hu et al. 2007). Based on our results, for an interquartile range increase in maternal blood arsenic (1.3 μg/L), birth weight is estimated to decrease by 77.5 g (95% CI: –127.8, –27.3), which is comparable in magnitude with estimated effects of prenatal secondhand tobacco smoke on infant birth weight among nonsmoking mothers (25 to 90 g decrease based on a review of studies published through 1998) (Misra and Nguyen 1999).

Our study is consistent with much of the previous research on arsenic and birth outcomes. Prior studies have measured arsenic in maternal and cord blood (Guan et al. 2012; Remy et al. 2014; Xu et al. 2011), urine (Fei et al. 2013; Rahman et al. 2009), hair (Huyck et al. 2007), and drinking water (Hopenhayn et al. 2003; Yang et al. 2003). Despite the use of different exposure measures, inverse associations have consistently been reported with fetal growth parameters. In New Hampshire, where private wells are common, maternal urinary arsenic was inversely associated with birth weight (Fei et al. 2013), and town-level modeled groundwater arsenic was associated with town-level term low birth weight in a geospatial analysis (Shi et al. 2015). In Bangladesh, a steeper negative dose–response curve for arsenic with birth outcomes was estimated at the lower exposure range (< 100 μg/L in urine) than at higher exposures (Rahman et al. 2009). Associations with lower-level exposures were also observed in a Belgian population (Remy et al. 2014), where blood arsenic was even lower than in our study (cord blood geometric mean = 0.56 μg/L).

Oxidative stress, inflammation, and placental insufficiency may be mechanisms that account for these findings. Arsenic is a pro-oxidant (Flora 2011), and arsenic exposure has been associated with increased levels of oxidative stress biomarkers in the placenta and in pregnant women (Ahmed et al. 2011; Engström et al. 2010). Prenatal arsenic has also been associated with increased expression of genes involved in inflammation, apoptosis, and stress response in cord blood (Fry et al. 2007), which could contribute adversely to fetal growth. Placental insufficiency, either via oxidative stress or other mechanisms such as epigenetic modifications, may link prenatal arsenic exposure to reduced fetal growth. Higher arsenic concentrations and reduced birth weight were associated with increased expression of a gene whose protein product scavenges vascular endothelial growth factor and plays a role in inhibiting placental angiogenesis (Remy et al. 2014). Inhibited placental angiogenesis can impair nutrition and restrict growth. Arsenic may also modulate expression of two arsenic-related genes (*AQP9*, *ENPP2*) in the placenta that are involved in arsenic transport and regulation of angiogenesis, respectively (Fei et al. 2013). The hormone leptin, which regulates appetite and metabolism, may also be involved: Positive associations have recently been reported between prenatal arsenic and cord blood leptin (Gossai et al. 2015), and cord blood leptin was negatively associated with length and head circumference at birth, as well as with lower weight gain from birth to 4 months, in 136–197 infants in the ALSPAC (Avon Longitudinal Study of Parents and Children) cohort (Ong et al. 1999). Cord blood leptin was also associated with lower BMI *z*-score, lower height-for-age *z*-score, and shorter leg length in 3-year-old children in the Project Viva cohort (Mantzoros et al. 2009). Arsenic exposure, even at relatively low to moderate levels, has been associated with evidence of epigenetic effects including global DNA hypomethylation and gene-specific hypermethylation, which may also be involved in its toxic mechanism of action (Bailey and Fry 2014; Intarasunanont et al. 2012).

In our data, inverse associations between arsenic and birth outcomes appeared stronger among women with impaired glucose tolerance compared with women without, though effect estimates were less precise due to the small number of subjects with impaired glucose tolerance. In the presence of impaired maternal glucose tolerance, arsenic may limit fetal growth more than among women with normal glucose metabolism. It is known that maternal hyperglycemia increases fetal insulin, which stimulates growth, and therefore offspring of mothers with gestational diabetes have higher birth weights (Silverman et al. 1991). Further, there is evidence linking elevated arsenic exposure with increased risk of impaired glucose tolerance during pregnancy (Ettinger et al. 2009) and type 2 diabetes (Navas-Acien et al. 2008). Thus, one might expect impaired maternal glucose metabolism to reduce, rather than increase, the magnitude of the inverse association between arsenic and birth weight. On the other hand, low birth weight has been associated with subsequent risk of type 2 diabetes (Harder et al. 2009), suggesting a link between insulin resistance and fetal thinness. Others have proposed that insulin resistance in the fetus, which may be genetically determined and/or influenced by maternal hyperglycemia, could impair angiogenesis (Hattersley and Tooke 1999), thereby reducing fetal growth. It is also possible that prenatal arsenic exposure lowers infant birth weight via a mechanism that acts independently of glucose metabolism, such as oxidative stress, overriding any effect of insulin to increase body weight. Both indirect effects on the fetus via maternal impaired glucose tolerance and direct effects via fetal insulin resistance could confer metabolic dysfunction on the fetus and increase later risk of type 2 diabetes.

There are limitations to this study. The use of blood to characterize arsenic exposure may be imperfect due to the short half-life in blood (Pomroy et al. 1980); therefore, exposure misclassification is possible. However, with continuous and steady exposure, steady-state concentrations of blood arsenic may be reached (NRC 1999). We measured total arsenic, which includes inorganic and organic species that may vary in their toxicity. Lack of speciated data could result in exposure misclassification. However, new research suggests that organoarsenicals, previously thought to be nontoxic, as well as inorganic species, may be transformed endogenously in humans (Molin et al. 2015); this adds to the total potential toxic arsenic burden that would be captured by measurements of total arsenic. Further, organoarsenicals likely represent a small fraction of total arsenic exposure because consumption of seafood, a primary source of organic arsenic exposure, is low in this population (62% of mothers reported never consuming a 3- to 5-oz serving of fish during pregnancy). The small number of women with impaired glucose tolerance limits our power to examine glucose–arsenic interactions in further depth. Unmeasured and residual confounding by smoking, secondhand smoke, or maternal diet/nutritional status is possible. Residual confounding by manganese exposure is also possible given that blood manganese is under tight regulatory control and may not be sensitive enough to accurately represent subtle differences in exposure. Finally, we found no statistically significant interactions between arsenic and lead or manganese, although joint effects of these three metals are possible and should be evaluated in future studies.

There are several strengths including that this study is among the first to examine associations between low-level metal exposures and birth outcomes in the United States. The availability of biomarker data on arsenic, lead, and manganese made it possible to adjust for important co-exposures, which few other studies have done. We evaluated both maternal and umbilical cord blood as biomarkers of prenatal exposure. Our finding that maternal arsenic was more strongly associated with birth outcomes than infant arsenic is consistent with the two other published studies with both biomarkers available (Guan et al. 2012; Xu et al. 2011), suggesting that maternal blood arsenic may be a more informative biomarker.

Exposure to arsenic remains a significant public health concern, especially in light of recent evidence that common consumer products contribute to exposures among pregnant women and children. Elevated arsenic concentrations have been measured in rice, rice products, fruit juices, and infant formulas (Gilbert-Diamond et al. 2011; Jackson et al. 2012; Wilson et al. 2012). Given the potential for relatively common fetal and early childhood exposures to arsenic, our finding that prenatal arsenic, even at low to moderate levels, can adversely impact birth outcomes is of considerable public health importance.

## Conclusions

Inverse associations between prenatal arsenic exposures and birth outcomes were observed while controlling for co-exposure to lead and manganese. This is one of the first reports of environmental arsenic associations with birth outcomes in a healthy U.S. population. These findings have wide-reaching implications because they are more generalizable to the U.S. population than studies conducted in highly exposed populations.

## Supplemental Material

(171 KB) PDF
