# Supplementary material for: Prenatal Arsenic Exposure and Birth Outcomes among a Population Residing near a Mining-Related Superfund Site
Source: Environ Health Perspect. 2016 Feb 9;124(8):1308–15. doi: 10.1289/ehp.1510070 (PMC4977047; doi:10.1289/ehp.1510070)
Supplement: (171 KB) PDF [file ehp.1510070.s001.acco.pdf]

Environ Health Perspect
DOI: 10.1289/ehp.1510070

Note to readers with disabilities: *EHP* strives to ensure that all journal content is accessible to all readers. However, some figures and Supplemental Material published in *EHP* articles may not conform to 508 standards due to the complexity of the information being presented. If you need assistance accessing journal content, please contact ehp508@niehs.nih.gov. Our staff will work with you to assess and meet your accessibility needs within 3 working days.

Supplemental Material

Prenatal Arsenic Exposure and Birth Outcomes among a Population Residing near a Mining-Related Superfund Site

Birgit Claus Henn, Adrienne S. Ettinger, Marianne R. Hopkins, Rebecca Jim, Chitra Amarasiriwardena, David C. Christiani, Brent A. Coull, David C. Bellinger, and Robert O. Wright

Table of Contents

Table S1. Adjusted associations of blood arsenic with birth outcomes
Table S2. Results from adjusted regression models evaluating interactions of arsenic with lead and manganese
Table S3. Adjusted associations of maternal and cord blood arsenic with birth outcomes, with and without accounting for measurement error using inverse variance weighting

Table S1. Adjusted associations[a] of blood arsenic with birth outcomes

Exposure	Birth Weight (g)		Gestational Age (weeks)		Birth Weight for Gestational Age (z-score)		Head Circumference (cm)	
	n	Estimate (95% CI)[b]	n	Estimate (95% CI)[b]	n	Estimate (95% CI)[b]	n	Estimate (95% CI)[b]
**Maternal blood arsenic**								
Quartile 1[c]	151	0 (reference)	151	0 (reference)	151	0 (reference)	146	0 (reference)
Quartile 2	145	-20.0 (-124.7, 84.8)	145	0.02 (-0.27, 0.31)	145	-0.06 (-0.27, 0.16)	138	0.00 (-0.41, 0.41)
Quartile 3	151	-78.4 (-182.4, 25.7)	151	-0.16 (-0.45, 0.13)	151	-0.11 (-0.32, 0.10)	144	-0.44 (-0.85, -0.04)
Quartile 4	149	-142.5 (-252.9, -32.2)	149	-0.41 (-0.72, -0.11)	149	-0.21 (-0.43, 0.01)	146	-0.48 (-0.91, -0.05)
Per IQR increase	596	-77.5 (-127.8, -27.3)	596	-0.13 (-0.27, 0.01)	596	-0.14 (-0.24, -0.04)	574	-0.22 (-0.42, -0.03)
**Cord blood arsenic**								
Quartile 1[d]	139	0 (reference)	139	0 (reference)	139	0 (reference)	135	0 (reference)
Quartile 2	151	78.5 (-28.8, 185.8)	151	0.30 (0.00, 0.59)	151	0.12 (-0.10, 0.33)	142	0.34 (-0.08, 0.76)
Quartile 3	159	-4.5 (-110.1, 101.0)	159	0.17 (-0.12, 0.46)	159	-0.02 (-0.23, 0.19)	152	-0.05 (-0.46, 0.37)
Quartile 4	139	-34.6 (-146.1, 76.9)	139	0.09 (-0.21, 0.40)	139	-0.09 (-0.31, 0.13)	136	0.00 (-0.44, 0.43)
Per IQR increase	588	-25.1 (-61.5, 11.4)	588	0.01 (-0.09, 0.11)	588	-0.05 (-0.13, 0.02)	565	-0.04 (-0.18, 0.10)

a – Models adjusted for maternal blood Pb and Mn (smoothed), maternal age at child's birth (smoothed), infant sex, race/ethnicity, parity, smoking during pregnancy, maternal education, prenatal vitamin use, and maternal hemoglobin at delivery.

b – Effect estimates represent change in birth outcomes for (1) arsenic quartiles 2, 3, 4 compared to quartile 1, or (2) IQR increase in arsenic (continuous $\log_e$-transformed concentrations, scaled to the IQR).

c – Maternal blood arsenic quartile 1: < 0.97 µg/l, quartile 2: ≥ 0.97 to < 1.4 µg/l, quartile 3: ≥ 1.4 to < 2.3 µg/l, quartile 4: ≥ 2.3 µg/l. d – Cord blood arsenic quartile 1: < 1.8 µg/l, quartile 2: ≥ 1.8 to < 2.4 µg/l, quartile 3: ≥ 2.4 to < 3.4 µg/l, quartile 4: ≥ 3.4 µg/l.

Table S2. Results from adjusted regression models[a] evaluating interactions of arsenic with lead and manganese[b].

Model and Exposure	Birth Weight (g)		Gestational Age (weeks)		Birth Weight for Gestational Age (z-score)		Head Circumference (cm)	
	n	Estimate (95% CI)	n	Estimate (95% CI)	n	Estimate (95% CI)	n	Estimate (95% CI)
**Model 1**	596		596		596		574	
Log_e Maternal As, per IQR increase[c]		-80.0 (-130.5, -29.6)		-0.13 (-0.27, 0.01)		-0.14 (-0.25, -0.04)		-0.23 (-0.43, -0.03)
Log_e Maternal Pb		41.1 (-35.2, 117.5)		0.14 (-0.07, 0.35)		0.05 (-0.10, 0.20)		0.37 (0.07, 0.67)
Maternal As * Pb		31.9 (-43.8, 107.6) p=0.41		-0.03 (-0.24, 0.18) p=0.79		0.07 (-0.08, 0.22) p=0.39		0.10 (-0.20, 0.39) p=0.52
**Model 2**	596		596		596		574	
Log_e Maternal As, per IQR increase		-79.0 (-131.2, -26.8)		-0.13 (-0.27, 0.02)		-0.14 (-0.25, -0.04)		-0.26 (-0.46, -0.05)
Log_e Maternal Mn		56.7 (-67.6, 180.9)		0.05 (-0.30, 0.39)		0.10 (-0.15, 0.35)		-0.61 (-1.1, -0.11)
Maternal As * Mn		12.9 (-102.2, 128.0) p=0.83		-0.03 (-0.35, 0.29) p=0.87		0.02 (-0.21, 0.25) p=0.85		0.24 (-0.21, 0.70) p=0.29
**Model 3**	588		588		588		565	
Log_e Cord As, per IQR increase[c]		-24.5 (-61.5, 12.4)		0.00 (-0.10, 0.10)		-0.05 (-0.13, 0.02)		-0.03 (-0.18, 0.11)
Log_e Cord Pb		31.6 (-76.0, 139.1)		0.16 (-0.14, 0.46)		0.02 (-0.19, 0.24)		0.27 (-0.15, 0.69)
Cord As * Pb		5.7 (-54.0, 65.4) p=0.85		-0.05 (-0.22, 0.11) p=0.54		0.02 (-0.10, 0.14) p=0.74		0.04 (-0.19, 0.27) p=0.72
**Model 4**	588		588		588		565	
Log_e Cord As, per IQR increase		-28.2 (-65.6, 9.2)		0.01 (-0.10, 0.11)		-0.06 (-0.13, 0.02)		-0.04 (-0.19, 0.10)

Log_e Cord Mn		-146.7<br>(-324.8, 31.5)		-0.07<br>(-0.56, 0.42)		-0.31<br>(-0.67, 0.05)		-0.69<br>(-1.4, 0.01)
Cord As * Mn		39.0<br>(-61.5, 139.5)<br>p=0.45		0.00<br>(-0.28, 0.28)<br>p=0.99		0.09<br>(-0.11, 0.29)<br>p=0.39		0.07<br>(-0.32, 0.46)<br>p=0.74

a – Models adjusted for blood lead and manganese, mother's age at delivery, child sex, race/ethnicity, parity, smoking during pregnancy, maternal education, prenatal vitamin use, and maternal hemoglobin at delivery. Lead and manganese centered at mean of log_e distribution.

b – One interaction term included in each model.

c – Continuous log_e-transformed arsenic concentrations, scaled to the IQR.

Table S3. Adjusted associations of maternal and cord blood arsenic with birth outcomes[a], with and without accounting for measurement error using inverse variance weighting

**Exposure**	**Birth Weight (g)**	**Gestational Age (weeks)**	**Birth Weight for Gestational Age (z-score)**	**Head Circumference (cm)**
	**Estimate (95% CI)[b]**	**Estimate (95% CI)[b]**	**Estimate (95% CI)[b]**	**Estimate (95% CI)[b]**
Log_e maternal As	-77.5 (-127.8, -27.3)	-0.13 (-0.27, 0.01)	-0.14 (-0.24, -0.04)	-0.22 (-0.42, -0.03)
Weighted log_e maternal As[c]	-77.1 (-126.7, -27.5)	-0.16 (-0.31, -0.02)	-0.13 (-0.23, -0.03)	-0.27 (-0.47, -0.08)
Log_e cord As	-25.1 (-61.5, 11.4)	0.01 (-0.09, 0.11)	-0.05 (-0.13, 0.02)	-0.04 (-0.18, 0.10)
Weighted log_e cord As[d]	-14.7 (-49.5, 20.1)	0.10 (0.01, 0.20)	-0.05 (-0.12, 0.02)	-0.01 (-0.12, 0.14)

a – Each line in table represents separate model. Models include blood lead and manganese, mother's age at delivery, child sex, race/ethnicity, parity, smoking during pregnancy, maternal education, prenatal vitamin use, and maternal hemoglobin at delivery.

b – Effect estimates represent change in birth outcomes per IQR increase in arsenic (continuous log_e-transformed concentrations, scaled to the IQR).

c – Weighted by the inverse of the variance of the maternal blood arsenic measurement.

d – Weighted by the inverse of the variance of the umbilical cord blood arsenic measurement.
